# Spatio‐Temporal Parameters of Endosomal Signaling in Cancer: Implications for New Treatment Options

**DOI:** 10.1002/jcb.25418

**Published:** 2015-11-16

**Authors:** Taras Stasyk, Lukas A. Huber

**Affiliations:** ^1^ BiocenterDivision of Cell BiologyInnsbruck Medical UniversityAustria; ^2^ ADSI – Austrian Drug Screening InstituteInnsbruckAustria

**Keywords:** ENDOSOME, LYSOSOME, SIGNALING, SCAFFOLDS, CANCER

## Abstract

The endo/lysosomal system in cells provides membranous platforms to assemble specific signaling complexes and to terminate signal transduction, thus, is essential for physiological signaling. Endocytic organelles can significantly extend signaling of activated cell surface receptors, and may additionally provide distinct locations for the generation of specific signaling outputs. Failures of regulation at different levels of endocytosis, recycling, degradation as well as aberrations in specific endo/lysosomal signaling pathways, such as mTORC1, might lead to different diseases including cancer. Therefore, a better understanding of spatio‐temporal compartmentalization of sub‐cellular signaling might provide an opportunity to interfere with aberrant signal transduction in pathological processes by novel combinatorial therapeutic approaches. J. Cell. Biochem. 117: 836–843, 2016. © 2015 The Authors. *Journal of Cellular Biochemistry* Published by Wiley Periodicals Inc.

**Abbreviations**
ARL8B: ADP‐ribosylation factor‐Like 8B
BMPR: bone morphogenetic protein receptor
EGFR: epidermal growth factor receptor
GEF: guanine nucleotide exchange factor
GLP‐1R: glucagon‐like peptide‐1 receptor
GPCRs: G protein—coupled receptors
HGF: hepatocyte growth factor
IQGAP1: IQ motif containing GTPase activating protein 1
LAMTOR: late endosomal/lysosomal adaptor and MAPK and mTOR activator
Met: hepatocyte growth factor receptor
MVB: multivesicular bodies
mTOR: mechanistic target of rapamycin kinase
mTORC1: mTOR kinase complex 1
PDGFR: platelet‐derived growth factor receptor
PAC1R: pituitary adenylate cyclase 1 receptor
PTHR: parathyroid hormone receptor type
RTKs: receptor tyrosine kinases
STAT3: signal transducer and activator of transcription 3
TGF: transforming growth factor
TSC: tuberous sclerosis complex
TSHR: thyroid‐stimulating hormone receptor
V2R: vasopressin type 2 receptor

Cells respond to different environmental stimuli by initiation of signal transduction at the plasma membrane. The binding of ligands to specific receptors at the cell surface, such as Receptor Tyrosine Kinases (RTKs) or G protein—coupled receptors (GPCRs), triggers the activation of many downstream signaling pathways, which are important for normal tissue homeostasis. For integration and regulation of complex signaling networks the endo/lysosomal system is essential [Miaczynska et al., 2004; Gould and Lippincott‐Schwartz, 2009; Sorkin and von Zastrow, 2009; Platta and Stenmark, 2011; Palfy et al., 2012]. Endosomes contribute to regulation of signal transduction in several different ways to ensure the appropriate signaling output in the proper time and the right place. The first well established type is regulation of the duration of cell surface receptor signaling and the number of exposed receptors by internalization and recycling/degradation. Secondly, upon internalization many cell surface receptors travel together with ligands in their activated state. Therefore, the first and usually short wave of signaling from the plasma membrane can be potentiated during receptor endocytosis before final termination of signal transduction by degradation in lysosomes. Importantly, this type of signaling from endosomes is not just a temporal extension of plasma membrane signaling, but adds additional signaling quality. Traveling endosomes provide access to new substrates with specific subcellular localization, which might lead to distinct physiological output. Often scaffolds and adaptor proteins are involved in organizing specific signaling units for endocytosed receptors *en route* to lysosomes. By a third mechanism, signaling components are exclusively localized to endosomes/lysosomes but not directly connected to receptor endocytosis.

The general principle of spatio‐temporal regulation of receptor‐mediated signal transduction is that the cellular response to different types of ligands is maximal and transient at the plasma membrane and sustained in endosomes. In this review we try to look at cellular signaling from an organelle‐centric point of view and present selected examples of deregulation of endosomal signaling in disease progression such as cancer. Hence, we discuss also perspectives in development of combinatorial therapies based on our current knowledge on compartmentalized signal transduction.

## INTERNALIZATION AND RECYCLING

Endocytosis of cell surface receptors is one of the control mechanisms of signal transduction initiated by extracellular stimuli. For a long time it has been assumed that receptors signal from the plasma membrane until they are internalized, endocytosed and sent to lysosomes for degradation. Stimulation of cells in vitro with appropriate ligands as, for instance, EGF reaches maximal EGFR activation levels within the first minutes of stimulation [Stasyk et al., 2007]. Ligand‐induced receptor signaling is tightly controlled by the rapid removal of receptors from the plasma membrane, which is the major regulator of signaling intensity. Once internalized, receptors can be transported through endosomal compartments either to lysosomes for degradation or they can be recycled back to the cell surface via recycling endosomes. Many activated receptors are detected in peripheral early endosomes at 10–30 min and reach perinuclear late endosomal compartments after 20–60 min upon ligand binding. An imbalance in receptor recycling might lead to sustained activation of receptors and could thereby promote transformation. Interestingly, the further destination of internalized receptors can differ, depending on the abundance of ligands as it was shown for EGFR. At low EGF doses the EGFR is recycled, but sent for lysosomal degradation at high ligand concentrations, thereby preventing overstimulation of cells [Sigismund et al., 2008].

Notably, different ligands can have diverse effects on recycling of the same receptor. Again the EGFR is a well‐established example for this. The receptor is directed for lysosomal degradation if induced by EGF but is recycled upon transforming growth factor (TGF)‐α stimulation. TGF‐α leads to sustained EGFR signaling and, therefore, is more mitogenic than EGF [Waterman et al., 1998]. Additionally, heparin‐binding EGF‐like growth factor and betacellulin target EGFR for lysosomal degradation, but in contrast epiregulin and amphiregulin lead to receptor recycling, similarly to TGF‐α [Roepstorff et al., 2009]. Many of these EGFR ligands are often upregulated in cancer due to the autocrine nature; therefore it was proposed that the oncogenic potential of different ligands depends on their ability to induce receptor recycling [Roepstorff et al., 2009]. A sustained stimulation with ligands that do not promote receptor down‐regulation but enhance recycling might be a general mechanism of constitutive proliferation in cancer, in addition to receptor overexpression as a result of gene amplification.

## RECEPTOR SIGNALING *EN ROUTE* TO LYSOSOMES

Signaling from endosomes has been demonstrated for a number of cell surface receptors from different receptor families such as the RTKs (e.g., EGFR, Met, PDGFR, and the insulin receptor), serine/threonine kinase receptors (transforming growth factor‐β (TGF‐β), the bone morphogenetic protein (BMP) and the activin receptors), GPCRs, toll‐like receptors, as well as interferon, Wnt and Notch receptors. Endosomal signaling of these receptors is well characterized and was extensively reviewed elsewhere [Hupalowska and Miaczynska, 2012; Barrow‐McGee and Kermorgant, 2014; Vilardaga et al., 2014; Tsvetanova et al., 2015]. Detailed analysis of different receptors is out of the scope of this review, only selected and very recent findings will be briefly discussed here.

There are several important characteristics of endosomal signaling that is spatially and temporally separated from signaling at the plasma membrane: 1) signaling complexes on organelles are different from those at the plasma membrane; 2) receptor endocytosis and active signaling from organelles are required for the full activation of their downstream effectors; and 3) there could be specific targets or distinct pathways stimulated by the same receptor depending on which endosome it is localized. These properties of endosomal signaling were very recently shown for Hepatocyte growth factor (HGF) receptor (Met) signaling from two different populations of endosomes in human breast cancer models [Menard et al., 2014]. Met signaling is initiated by HGF at the plasma membrane of epithelial and endothelial cells. It was shown previously that the small GTPase Rac1, which acts downstream of Met, in a key pathway controlling cell migration, is activated on endosomes to trigger actin cytoskeleton reorganization at the plasma membrane [Palamidessi et al., 2008]. Kermogant and colleagues described two distinct pathways stimulated by Met depending on which organelles, that is, peripheral early or perinuclear late endosomes, the receptor is localized [Menard et al., 2014]. Remarkably, to stimulate breast cancer cell migration and invasion Rac1, has to be activated from late endosomes, where PI3K and guanine nucleotide exchange factor (GEF) VAV2 are specifically engaged. However, they are not required for an acute activation of Rac1 from early endosomes.

Scaffolds and adaptor proteins often organize specific endosomal signaling complexes. For example, such complexes containing activated endocytosed EGFR are organized on early endosomes by APPL, which recruits AKT and its substrate GSK3 [Miaczynska et al., 2004] and by the LAMTOR2/3 (p14/MP1) scaffold complex on late endosomes, which engages MEK1 and ERK1/2 [Teis et al., 2002]. Such scaffolding proteins can bind and organize multiple signaling proteins in a complex by non‐catalytic protein–protein interactions. Hence, trafficking and subcellular localization of MAPK signaling complexes within the cell can dictate the biological response [Taub et al., 2007]. Thereby, the duration of the signal does influence the nature of the biological response. Thereby cells use a rather simple regulatory principle to control complex and highly specific biological responses during MAPK signaling. Not surprisingly, loss of this fine tuned control of temporal or spatial regulation of MAPK signaling by mutations or changes in expression of scaffold proteins regulating MAPK signaling can make a significant contribution to many different diseases, including infection, immunosupression, and cancer [Pawson, 2004; Teis et al., 2006; Bohn et al., 2007].

Another well‐established example is activated transforming growth factor‐β receptor (TGFβR), which upon internalization interacts with Smad anchor for receptor activation (SARA) on early endosomes. In case of bone morphogenetic protein receptor (BMPR) another specific scaffold, endofin, organizes signaling from endosomes. These and many other scaffolds that make possible activation of their downstream targets at specific subcellular locations, were previously comprehensively summarized in [Palfy et al., 2012].

An interesting mechanism of early endosomal EGFR signaling, sustained by the non‐receptor tyrosine kinase PTK2B/PYK2, was reported recently [Verma et al., 2015]. Upon EGF induced phosphorylation PYK2 translocates to early endosomes and co‐localizes there with EGFR from where it enhances cell migration and potentiates epithelial‐to‐mesenchymal transition (EMT) in human breast carcinoma. It was proposed in this publication that PYK2 links EGF‐induced STAT3 phosphorylation followed by profound PYK2 transcription activation as well as partially induced Met expression as a positive feedback loop, which prolongs signaling and potentiates EMT. These results suggest formation of a specific early endosomal signaling complex, consisting of EGFR, PYK2 and pSTAT3, in response to EGF treatment. Interestingly, PYK2 depletion facilitates lysosomal targeting and degradation of EGFR. Moreover, PYK2 expression was found to correlate with high tumor grade and metastasis formation in patients, demonstrating possible therapeutic implications of the disruption of this endosomal‐signaling cascade.

Recent data suggest that GPCRs mediate the production of cyclic AMP not only from the plasma membrane but also from endosomal membranes. Originally, two studies have shown that two GPCRs, thyroid‐stimulating hormone receptor (TSHR) [Calebiro et al., 2009] and parathyroid hormone receptor type 1 (PTHR) [Ferrandon et al., 2009], continue to stimulate cAMP production in a sustained manner after internalization and their redistribution in Rab5‐positive endosomes. Endosomal cAMP generation has been further described for several GPCRs, such as the D1 dopamine receptor, the pituitary adenylate cyclase 1 receptor (PAC1R), the glucagon‐like peptide‐1 receptor (GLP‐1R), the vasopressin type 2 receptor (V2R), for reviews see [Vilardaga et al., 2014; Tsvetanova et al., 2015]. The current model proposes that activated cell surface GPCRs redistribute into early endosomes, from where signaling can be extended. Direct evidence that GPCRs and cognate G protein activation indeed occurs in endosomes came from recent experiments employing nanobody‐based biosensors in living mammalian cells [Irannejad et al., 2013]. In this report von Zastrow and colleagues used conformational active‐state‐specific single‐domain antibodies and detected two temporally and spatially separated waves of β2AR signaling and its associated Gs protein. The first one was detected within 2 min after agonist application at the cell membrane and involves ligand‐receptor interaction with the Gs protein. The second wave occurs on early endosomes after receptor internalization. Both signaling waves led to the accumulation of cAMP by the enzyme adenylyl cyclase, reaching a maximum of two signaling waves within approximately 10 min. Interestingly, the second discrete phase of β2AR signaling is separated from the first wave by an endocytosis event and appears to begin shortly after the delivery of receptors to early endosomes [Irannejad et al., 2013].

## ENDOCYTOSIS INDEPENDENT ENDO/LYSOSOMAL SIGNALING

All examples discussed above, of specific endosomal signaling complexes, contain internalized and endocytosed receptors of different families and scaffolds specific for different receptors and (sub‐)populations of endosomes. In addition to this well established and commonly accepted type of endosomal signaling there is an emerging field of organelle signaling independent from endocytosis, if the latter one is defined as active transportation of cell surface components into the cell. In most eukaryotic cells the plasma membrane makes up only for a minor part of all cellular membranes. The endo/lysosomal system, as a component of cellular endomembranes, provides membranous platforms to assemble specific signaling complexes at specific subcellular locations. Here we will briefly discuss endolysosomal signaling of the mechanistic (also known as mammalian) target of rapamycin (mTOR) kinase.

Endolysosomes are the fusion product between late endosomes and lysosomes and the place where most of the hydrolysis of endocytosed cargo takes place [Huotari and Helenius, 2011]. Endolysosomes function as signaling platforms in activating the mTOR kinase complex 1 (mTORC1) in response to nutrients and growth factors. MTORC1 is a highly conserved activator of cell growth, which is regulated by a variety of growth factors, cytokines and hormones, including insulin and insulin‐like growth factor [Dibble and Manning, 2013]. Hyperactivation of mTORC1 signaling is associated with human pathologies including diabetes and cancer [Zoncu et al., 2011]. Interestingly, the LAMTOR complex mentioned above as late endosomal scaffold for EGFR signaling is also absolutely required for mTORC1 signaling, which is activated primarily on the endolysosome. The LAMTOR complex (also known as Ragulator [Sancak et al., 2010]) consists of five proteins: p18/p14/MP1/C7orf59/HBXIP (LAMTOR1/2/3/4/5, respectively) and serves as a GEF to the heterodimeric RagA/B‐RagC/D GTPases, thereby mediating the translocation of mTORC1 to the lysosomal surface. We and others have shown recently that the LAMTOR complex together with a lysosomal transporter SLC38A9 can sense amino acid levels that control the activation of mTOR on lysosomes [Rebsamen et al., 2015; Wang et al., 2015; Wolfson et al., 2015]. The importance of the subcellular localization in the regulation of mTORC1 signaling was demonstrated recently by two groups Menon et al. [2014] and Demetriades et al. [2014], who showed that mTORC1 deactivation is determined by recruitment of the tuberous sclerosis complex TSC to the lysosome. Interestingly, presence of the LAMTOR complex on lysosomes, late endosomes and sub‐population of intermediate multivesicular bodies (MVB), with characteristics of both late and early endosomes [Vogel et al., 2015] might suggest different subcellular locations of mTORC1.

## ENDOSOMES AS MOBILE SIGNALING VEHICLES

It has become evident already some time ago that endosomes play a role in cell migration [Sadowski et al., 2009; Schiefermeier et al., 2011]. We have recently shown that late endosomes can transport signaling complexes towards the cell periphery to promote cell migration. A specific subpopulation of the Rab7‐positive endosomes, which carry the LAMTOR scaffold complex, can move from the cell center along microtubules towards the cell periphery. There they target the dynamic regions of mature focal adhesions and thereby stimulate focal adhesion turnover that is necessary for cell migration [Schiefermeier et al., 2014]. Directed organelle motility is kinesin‐dependent, because knocking down of the small GTPase Arl8B, known to recruit the motor protein kinesin‐1 to late endosomes, abrogated the delivery of late endosomes to focal adhesions and subsequently impaired cell migration. This study also suggested a possible mechanism of late endosomal stimulation of cell migration by promoting the removal of the IQGAP1 from mature adhesions.

Another interesting example of endosomes as vehicles of signals over long distance is transportation of activated STAT3 from the plasma membrane into the nucleus upon activation with EGF and PDGF [Bild et al., 2002] or HGF [Kermorgant and Parker, 2008]. STAT3 co‐localizes with activated and internalized receptors in endocytic vesicles on their way from the plasma membrane to the perinuclear region, but the activation of STAT3 is low because of cytoplasmic phosphatases action that does not allow pSTAT3 diffusion and nuclear uptake. STAT3 gets only effectively phosphorylated and accumulates in the nucleus upon delivery to late endosomes at the perinuclear compartment where a sufficient phosphorylation/dephosphorylation threshold is achieved.

Above‐mentioned examples from different fields compose quite a significant body of evidence for spatial and temporal consequences of endosomal signaling. It is particularly exciting task for the future to demonstrate different physiological responses produced by activated receptors at the cell membrane and at endosomes. Better understanding of how to specifically interfere with the two waves of signaling might be beneficial for therapeutic purposes to treat different diseases, including cancer.

## ENDOCYTIC SIGNALING IN CANCER

Deregulations of signal transduction at different subcellular levels, caused by defective trafficking of growth factor receptors, can drive tumorigenesis (Table I). Mutations in receptors that make them constitutively active, impaired receptor‐mediated endocytosis, such as increased recycling or decreased degradation, mislocalization of active signaling complexes as well as mutations of negative regulators can all contribute to the pathogenesis of cancer [Mellman and Yarden, 2013].

**Table I:** Selected Examples of Oncogenic Mutations in Endo/Lysosomal Signaling

Protein/complex	Mutation	Signaling from organelle	References
I. Surface receptors internalization			
Dynamin	Upregulated in pancreatic cancer, a potent activator of metastatic migration	GTPase responsible for the scission of newly formed endocytic vesicles	Razidlo et al. [2013]
PHD3 (prolyl hydroxylase domain protein 3)	Loss suppresses ​EGFR internalization and hyperactivates ​EGFR signaling	Scaffolding protein that promotes the internalization of ​EGFR	Henze et al. [2014]
II. Recycling endosomes			
HER2	Overexpression	Enhances recycling of EGFR‐HER2 heterodimers	Worthylake et al. [1999]
NDRG1 (N‐myc down regulated gene1)	Downregulation increases prostate and breast metastasis	Localizes to the endosomes and is a Rab4a effector involved in vesicular recycling	Bandyopadhyay et al. [2003]
III. Early endosomes			
PYK2	High expression in many human tumors, correlate with tumor grade and lymph node metastasis	Sustains early endosomal EGFR signaling, enhances cell migration and EMT	Verma et al. [2015]
Beclin1	Tumor suppressor that is decreased in many human tumors which enhance breast cancer progression	Impaired early endosome maturation, dysregulation of growth factor receptor signaling and autophagy	Rohatgi et al. [2015]
IV. Late endosomes			
Met	M1268T and D1246N in the kinase domain of Met	Sustains late endosomal Rac1 signaling, triggers cell migration and invasion	Joffre et al. [2011]
V. Lysosomes			
mTORC1	Hyper‐activation of upstream activators PI3K, Akt, RAS, RAF; Rheb overexpression; mutations or genetic loss of tumor suppressor: TSC1/2, DEPTOR, PTEN, p53, LKB1, NF1	A highly conserved activator of cell growth, recruited by LAMTOR/Ragulator‐Rag complex specifically to late endosomes and lysosomes in response to nutrients and growth factors	Zoncu et al. [2011], Hoogeveen‐Westerveld et al. [2013]

One well‐established oncogenic deregulation of cell surface receptors is their escape from ligand‐stimulated ubiquitination by the Cbl family of adaptor proteins and ligases that function as negative regulators of many signaling pathways. Such mechanism was described for EGFRvIII, which is the most common mutation of EGFR gene. This tumor‐specific deletion in the extracellular domain of EGFR contributes to the formation of many epithelial malignancies. In some brain tumors this mutation of EGFR occurs at extremely high frequency. Interestingly, although EGFRvIII possesses only about 10% of the intrinsic activity of wild‐type EGFR its net signaling rate is enhanced by the delay of its endocytosis. The low level of EGFRvIII signaling causes hypo‐phosphorylation of the receptor that prevents engaging of Cbl and, therefore reduced polyubiquitination and degradation of the receptor, and, therefore, increased recycling. This may also play a critical role in the development of resistance to tyrosine kinase inhibitor treatments as observed in the clinics [Han et al., 2006].

Aberrant activity of GPCRs is frequently associated with tumorigenesis. Recent deep sequencing studies show that GPCRs are mutated in nearly 20% of human cancers [O'Hayre et al., 2013]. Activating mutations in TSHR receptors with known endosomal signaling, in approximately 30% of thyroid cancers, strongly suggest a link between mutated GPCR signaling and human cancer. Consistent with the role for GPCRs in tumor growth, constitutively active mutants of G proteins have also been identified. For example, more than 4 % of tumors were shown to carry activating mutations in Gs, but in some specific tumor types, like in the case of pancreatic adenocarcinoma, Gs mutations are found in 66% of intraductal papillary mucinous neoplasms [O'Hayre et al., 2013]. In vivo tumorigenicity of oncogenic Met mutants was demonstrated to be caused by their accumulation and signaling on endosomes, therefore, directly linking RTK endocytosis and cancer development. Constitutively active Met, mutated in the kinase domain (M1268T or D1246N), exhibits increased recycling and decreased degradation, leading to accumulation on endosomes and, therefore, sustained activation of the Rac1, enhanced cell migration and metastasis [Joffre et al., 2011].

Upregulation of lysosomal mTORC1 signaling is one of the most common hallmarks of human cancers [Zoncu et al., 2011]. Hyper‐activation of upstream activators such as PI3K, Akt or Rheb has been observed in many types of tumors. On the other side, mutations or genetic loss of upstream negative suppressors of mTORC1 signaling, including TSC (tuberous sclerosis complex) and PTEN (phosphatase and tensin homologue), can also cause tumor syndromes. More than one hundred mutations were detected in either TSC1 or TSC2, which cause the Tuberous Sclerosis complex, an autosomal disease characterized by the formation of hamartomas in several tissues [Hoogeveen‐Westerveld et al., 2013]. Another endogenous mTOR inhibitor DEPTOR (DEP domain containing mTOR‐interacting protein) was found to be expressed at low levels in most cancers. Hyper‐activation of the mTORC1 signaling contributes significantly to cancer development, therefore, targeting of the mTORC1 pathway at different levels and different components, including activators, inhibitors and adaptors, could potentially be an effective therapeutic option. Along those lines, upon genetic ablation of the LAMTOR2 protein in dendritic cells, the entire Ragulator/LAMTOR complex gets destabilized on late endosomes and as a consequence signaling is uncoupled: MAPK signaling goes down, mTOR signaling is enhanced. The consequences of such uncoupled signaling, involving two of the major signaling pathways regulating cell growth and proliferation, are severe. While ERK signaling is abolished the elevated mTOR signaling at the same time induces a massive expansion of pre‐DCs and DCs resulting in a myeloid proliferative disorder. As a possible mechanism we could identify a transport defect of the Flt3 receptor to the lysosome, followed by an increase of the receptor on the surface of DCs. Interestingly, rapamycin and ACC220 could treat this myeloid proliferative disorder in mice, which provides new therapeutic windows of opportunity in the treatment of myeloid proliferative disorders in general [Scheffler et al., 2014].

## COMBINATORIAL THERAPY AND FUTURE PROSPECTS

**Figure 1:**
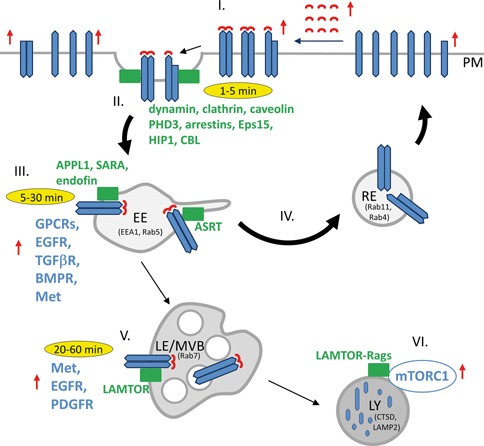
Druggable points of oncogenic endo/lysosomal signaling. Receptor‐mediated signal transduction is deregulated in human tumors at different levels of receptor activation and trafficking, labeled here with red arrows. Oncogenic mutations in different receptors result in increased recycling and in a decrease in the degradation of the receptors. Increased recycling of activated receptors is delineated in the scheme with bold arrows. In addition to conventional anticancer therapies employing, e.g., tyrosine kinase inhibitors (I), novel combinatorial approaches could interfere with endo/lysosomal signaling and/or aberrant receptor trafficking at the levels of receptor internalization (II), early endosomal signaling (III), receptor recycling back to the plasma membrane via recycling endosomes (IV), signaling from late endosomes (V) or lysosomes (VI). Endosomal adaptor and scaffold proteins, which organize signaling complexes in the organelles and molecular sorting machineries that determine receptor trafficking (shown in green) could be potential anticancer targets. ASRT, actin‐sorting nexin 27‐retromer tubule; EE, early endosome; LE/MVB, late endosome/multivesicular body; LY, lysosome; PM, plasma membrane; RE, recycling endosome.

Since patients often do not respond to conventional anti‐cancer drugs or develop resistance, alternative therapies are required. According to the current sequential protocol, the patient is administered one by one to different drugs, for example the tyrosine kinase inhibitors, until the optimal response is reached. Unfortunately the risk of multidrug resistance in this approach is increased. An alternative therapeutic approach by administering combinations of different drugs at the same time could provide a cure for patients that did otherwise not respond to conventional therapies. Appropriate combinations should potentially enhance response and decrease development of drug resistance. Combinations of well‐established drugs, which inhibit receptors on the cell surface with specific inhibitors of endosomal signaling as well as more general inhibitors of endocytosis, which act on different levels of the process such as internalization, recycling or degradation (see Fig. 1) might open new therapeutic opportunities in the future. Some inhibitors of endocytosis are being explored as potential anticancer drugs. For example dynoles, dynamin GTPase inhibitors, have a potentially promising therapeutic window as antiproliferative agents against cancer cells (Table II). Dyngo‐4a, another dynamin inhibitor, specifically and significantly reduced the second endosomal phase of β2AR signaling, which is one of the prototypic GPCRs, but did not detectably affect signaling at the plasma membrane [Irannejad et al., 2013]. As GPCRs are the target of more than 25% of all drugs on the market, novel therapeutic strategies aimed at specific targeting of endosomal GPCR signaling or receptor trafficking in general could be beneficial for cancer treatment.

**Table II:** Examples of Anti‐Cancer Drugs Applicable for Endo/Lysosomal Signaling for Possible Combinatorial Approaches (Modified From Hojjat‐Farsangi [2014], Vilardaga et al. [2014], and von Kleist and Haucke [2012])

Targets	Inhibitors	Mechanism
RTKs	FDA approved RTK inhibitors	
HER2, EGFR	Afatinib, Lapatinib	A small molecule dual tyrosine kinase inhibitors
EGFR	Erlotinib, Gefitinib, Icotinib	Reversible tyrosine kinase inhibitors
VEGFR	Lenvatinib	Inhibits both VEGFR2 and VEGFR3 kinases
Met	Crizotinib	A small‐molecule dual inhibitor of the c‐Met and ALK
Endocytic targets		
Dynamin	Dynasore, dynoles, dyngoes, Bis‐T	GTPase inhibitors that target dynamin‐dependent endocytosis
	MitMAB, OctMAB	Block dynamin association with lipids
Clathrin	Pitstop 2	Clathrin‐mediated endocytosis
PIP5KIII (PIKfyve)	YM201636	Inhibitor of PIKfyve‐catalyzed PtdIns(3,5)P2 synthesis, disrupts late endosomal/lysosomal and autophagosomal fusion
mTORC1	Rapamycin and FDA approved rapalogs (Temsirolimus, Everolimus)	The potent natural antibiotic, rapamycin, or its derivatives form a complex with FKBP12 protein, which then binds directly to mammalian TORC1

Met signaling from late endosomes was recently shown to be crucial for sustained Rac1 activation in breast cancer cells, responsible for cell spreading and metastasis formation [Menard et al., 2014]. Therefore, combination of conventional anti‐cancer treatment with more specific interference with Met targeting to late endosomes, formation of specific signaling complexes on late endosomes or their activation from late endosomes might possibly be an alternative strategy in the future cancer therapies in addition to specific anti Met drugs being tested now on patients in clinical trials. This possibility was originally demonstrated on HGF/Met signaling dependent tumor progression, stimulated by two distinct activating mutations in the kinase domain of Met [Joffre et al., 2011] and shown recently also in a hepatocellular carcinoma (HCC) model [Hu et al., 2015]. HGF‐induced intrahepatic metastasis in mice, injected with the human hepatoma cell line HepG2, were prevented by Dynasore, the inhibitor of dynamin and endocytosis, suggesting novel therapeutic endosomal targets for the treatment of HGF‐induced HCC.

Another combinatorial approach could be the simultaneous inhibition of several receptors or the targeting of common signaling pathways, in the context of crosstalk between different receptors. As discussed above, EGFinduced early endosomal PYK2 signaling, which leads to PYK2‐STAT3‐Met positive feedback [Verma et al., 2015]. This would suggest that combinatorial treatment, including EGFR‐ and Met‐ as well as PYK2‐inhibitors, might be highly synergistic against certain breast cancer subtypes. Additionally, inhibition of several different receptors by inhibition of recycling in combination with simultaneous activation of endocytosis might have synergistic therapeutic potential.

All those inhibitors of endocytosis are still pretty far from preclinical development since they might target too common cellular mechanisms with potentially high toxicity at the organism level. However, the mode of action with targeting endocytosis would suggest that chemical libraries could be developed in high‐contents screening setups that act like molecular chaperons by selectively only disturbing endocytosis and recycling of certain mutated oncogenic receptors.

Margalef et al. have shown recently that inhibition of the endosomal V‐ATPase is a potential therapeutic strategy for the treatment of colorectal cancers with mutant BRAF, which is predictive of poor prognosis and therapeutic resistance [Margalef et al., 2015]. A new approach is based on decreasing the acidity of endosomes and therefore inhibition of the P45‐IKKα activation, an endosomal protein that needs a specific acidic environment and is essential for tumor progression. The authors found that the inhibition of acidification of endosomes induced tumor cell death in culture and enhanced the effect of conventional chemotherapy in mice leading to suppression of colon tumor growth and metastasis in mice. Interestingly, proton pump inhibitors are common therapeutic reagents for reflux esophagitis and *H. pylori* eradication in the clinics and have also been used recently in tumor models. Proton pump inhibitor pretreatment inhibited V‐type H^+^‐ATPase activity and increased both extracellular pH and pH of lysosomal organelles. Hence, in human/mouse xenograft models oral pretreatment with proton pump inhibitors is able to sensitize human solid tumors to anticancer drugs [De Milito and Fais, 2005].

An approach combining the mTORC1 inhibitor Everolimus with an aromatase inhibitor improved progression‐free survival in patients with hormone‐receptor‐positive advanced breast cancer [Baselga et al., 2012] and stimulated new pre‐clinical tests and clinical trials. Important for the spatial regulation of endosomal signaling, we are addressing in this review, is a finding described a few years ago that lysosomal positioning regulates the protein kinase complex mTORC1 activity [Korolchuk et al., 2011]. It was shown in this study that lysosomes move more closely to the plasma membrane in response to nutrient availability, whereas starvation causes perinuclear clustering of the organelles facilitating autophagy. Particularly, overexpression or knockdown of the kinesin motor protein KIF2 or the late endosomal/lysosomal small GTPase Arl8B regulated lysosomal distribution and mTORC1 activity. Taking into account that increased mTORC1 activity accompanied by decreased autophagy is strongly associated with tumorigenesis, as well as that GTPases are potentially druggable targets, this finding may have therapeutic potential for possible combinatorial approaches.

We anticipate that selective targeting of the endosomal signaling, especially in combination with conventional anti‐cancer therapy and targeted small molecules, may offer more effective treatments. The here suggested link between endosomal signaling and cancer increases expectations from this type of combinatorial therapeutics in future.

## References

[jcb25418-bib-0001] Bandyopadhyay S , Pai SK , Gross SC , Hirota S , Hosobe S , Miura K , Saito K , Commes T , Hayashi S , Watabe M , Watabe K . 2003 The Drg‐1 gene suppresses tumor metastasis in prostate cancer. Cancer Res 63:1731–1736. 12702552

[jcb25418-bib-0002] Barrow‐McGee R , Kermorgant S . 2014 Met endosomal signalling: In the right place, at the right time. Int J Biochem Cell Biol 49:69–74. 24440758 10.1016/j.biocel.2014.01.009

[jcb25418-bib-0003] Baselga J , Campone M , Piccart M , Burris HA, 3rd , Rugo HS , Sahmoud T , Noguchi S , Gnant M , Pritchard KI , Lebrun F , Beck JT , Ito Y , Yardley D , Deleu I , Perez A , Bachelot T , Vittori L , Xu Z , Mukhopadhyay P , Lebwohl D , Hortobagyi GN . 2012 Everolimus in postmenopausal hormone‐receptor‐positive advanced breast cancer. N Engl J Med 366:520–529. 22149876 10.1056/NEJMoa1109653 PMC5705195

[jcb25418-bib-0004] Bild AH , Turkson J , Jove R . 2002 Cytoplasmic transport of Stat3 by receptor‐mediated endocytosis. EMBO J 21:3255–3263. 12093727 10.1093/emboj/cdf351 PMC126099

[jcb25418-bib-0005] Bohn G , Allroth A , Brandes G , Thiel J , Glocker E , Schaffer AA , Rathinam C , Taub N , Teis D , Zeidler C , Dewey RA , Geffers R , Buer J , Huber LA , Welte K , Grimbacher B , Klein C . 2007 A novel human primary immunodeficiency syndrome caused by deficiency of the endosomal adaptor protein p14. Nat Med 13:38–45. 17195838 10.1038/nm1528

[jcb25418-bib-0006] Calebiro D , Nikolaev VO , Gagliani MC , de Filippis T , Dees C , Tacchetti C , Persani L , Lohse MJ . 2009 Persistent cAMP‐signals triggered by internalized G‐protein‐coupled receptors. PLoS Biol 7:e1000172. 19688034 10.1371/journal.pbio.1000172 PMC2718703

[jcb25418-bib-0007] De Milito A , Fais S . 2005 Tumor acidity, chemoresistance and proton pump inhibitors. Future Oncol 1:779–786. 16556057 10.2217/14796694.1.6.779

[jcb25418-bib-0008] Demetriades C , Doumpas N , Teleman AA . 2014 Regulation of TORC1 in response to amino acid starvation via lysosomal recruitment of TSC2. Cell 156:786–799. 24529380 10.1016/j.cell.2014.01.024 PMC4346203

[jcb25418-bib-0009] Dibble CC , Manning BD . 2013 Signal integration by mTORC1 coordinates nutrient input with biosynthetic output. Nat Cell Biol 15:555–564. 23728461 10.1038/ncb2763 PMC3743096

[jcb25418-bib-0010] Ferrandon S , Feinstein TN , Castro M , Wang B , Bouley R , Potts JT , Gardella TJ , Vilardaga JP . 2009 Sustained cyclic AMP production by parathyroid hormone receptor endocytosis. Nat Chem Biol 5:734–742. 19701185 10.1038/nchembio.206 PMC3032084

[jcb25418-bib-0011] Gould GW , Lippincott‐Schwartz J . 2009 New roles for endosomes: From vesicular carriers to multi‐purpose platforms. Nat Rev Mol Cell Biol 10:287–292. 19277045 10.1038/nrm2652 PMC3690957

[jcb25418-bib-0012] Han W , Zhang T , Yu H , Foulke JG , Tang CK . 2006 Hypophosphorylation of residue Y1045 leads to defective downregulation of EGFRvIII. Cancer Biol Ther 5:1361–1368. 16969069 10.4161/cbt.5.10.3226

[jcb25418-bib-0013] Henze AT , Garvalov BK , Seidel S , Cuesta AM , Ritter M , Filatova A , Foss F , Dopeso H , Essmann CL , Maxwell PH , Reifenberger G , Carmeliet P , Acker‐Palmer A , Acker T . 2014 Loss of PHD3 allows tumours to overcome hypoxic growth inhibition and sustain proliferation through EGFR. Nat Commun 5:5582. 25420773 10.1038/ncomms6582 PMC4263145

[jcb25418-bib-0014] Hojjat‐Farsangi M . 2014 Small‐molecule inhibitors of the receptor tyrosine kinases: Promising tools for targeted cancer therapies. Int J Mol Sci 15:13768–13801. 25110867 10.3390/ijms150813768 PMC4159824

[jcb25418-bib-0015] Hoogeveen‐Westerveld M , Ekong R , Povey S , Mayer K , Lannoy N , Elmslie F , Bebin M , Dies K , Thompson C , Sparagana SP , Davies P , van Eeghen AM , Thiele EA , van den Ouweland A , Halley D , Nellist M . 2013 Functional assessment of TSC2 variants identified in individuals with tuberous sclerosis complex. Hum Mutat 34:167–175. 22903760 10.1002/humu.22202

[jcb25418-bib-0016] Hu CT , Cheng CC , Wu JR , Pan SM , Wu WS . 2015 PKCepsilon‐mediated c‐Met endosomal processing directs fluctuant c‐Met‐JNK‐paxillin signaling for tumor progression of Hep G2. Cell Signal 27:1544–1555. 25778903 10.1016/j.cellsig.2015.02.031

[jcb25418-bib-0017] Huotari J , Helenius A . 2011 Endosome maturation. EMBO J 30:3481–3500. 21878991 10.1038/emboj.2011.286 PMC3181477

[jcb25418-bib-0018] Hupalowska A , Miaczynska M . 2012 The new faces of endocytosis in signaling. Traffic 13:9–18. 21752167 10.1111/j.1600-0854.2011.01249.x

[jcb25418-bib-0019] Irannejad R , Tomshine JC , Tomshine JR , Chevalier M , Mahoney JP , Steyaert J , Rasmussen SG , Sunahara RK , El‐Samad H , Huang B , von Zastrow M . 2013 Conformational biosensors reveal GPCR signalling from endosomes. Nature 495:534–538. 23515162 10.1038/nature12000 PMC3835555

[jcb25418-bib-0020] Joffre C , Barrow R , Menard L , Calleja V , Hart IR , Kermorgant S . 2011 A direct role for Met endocytosis in tumorigenesis. Nat Cell Biol 13:827–837. 21642981 10.1038/ncb2257

[jcb25418-bib-0021] Kermorgant S , Parker PJ . 2008 Receptor trafficking controls weak signal delivery: A strategy used by c‐Met for STAT3 nuclear accumulation. J Cell Biol 182:855–863. 18779368 10.1083/jcb.200806076 PMC2528569

[jcb25418-bib-0022] Korolchuk VI , Saiki S , Lichtenberg M , Siddiqi FH , Roberts EA , Imarisio S , Jahreiss L , Sarkar S , Futter M , Menzies FM , O'Kane CJ , Deretic V , Rubinsztein DC . 2011 Lysosomal positioning coordinates cellular nutrient responses. Nat Cell Biol 13:453–460. 21394080 10.1038/ncb2204 PMC3071334

[jcb25418-bib-0023] Margalef P , Colomer C , Villanueva A , Montagut C , Iglesias M , Bellosillo B , Salazar R , Martinez‐Iniesta M , Bigas A , Espinosa L . 2015 BRAF‐induced tumorigenesis is IKKalpha‐dependent but NF‐kappaB‐independent. Sci Signal 8:ra38. 25900832 10.1126/scisignal.2005886

[jcb25418-bib-0024] Mellman I , Yarden Y . 2013 Endocytosis and cancer. Cold Spring Harb Perspect Biol 5:a016949. 24296170 10.1101/cshperspect.a016949 PMC3839607

[jcb25418-bib-0025] Menard L , Parker PJ , Kermorgant S . 2014 Receptor tyrosine kinase c‐Met controls the cytoskeleton from different endosomes via different pathways. Nat Commun 5:3907. 24835487 10.1038/ncomms4907

[jcb25418-bib-0026] Menon S , Dibble CC , Talbott G , Hoxhaj G , Valvezan AJ , Takahashi H , Cantley LC , Manning BD . 2014 Spatial control of the TSC complex integrates insulin and nutrient regulation of mTORC1 at the lysosome. Cell 156:771–785. 24529379 10.1016/j.cell.2013.11.049 PMC4030681

[jcb25418-bib-0027] Miaczynska M , Christoforidis S , Giner A , Shevchenko A , Uttenweiler‐Joseph S , Habermann B , Wilm M , Parton RG , Zerial M . 2004 APPL proteins link Rab5 to nuclear signal transduction via an endosomal compartment. Cell 116:445–456. 15016378 10.1016/s0092-8674(04)00117-5

[jcb25418-bib-0028] O'Hayre M , Vazquez‐Prado J , Kufareva I , Stawiski EW , Handel TM , Seshagiri S , Gutkind JS . 2013 The emerging mutational landscape of G proteins and G‐protein‐coupled receptors in cancer. Nat Rev Cancer 13:412–424. 23640210 10.1038/nrc3521 PMC4068741

[jcb25418-bib-0029] Palamidessi A , Frittoli E , Garre M , Faretta M , Mione M , Testa I , Diaspro A , Lanzetti L , Scita G , Di Fiore PP . 2008 Endocytic trafficking of Rac is required for the spatial restriction of signaling in cell migration. Cell 134:135–147. 18614017 10.1016/j.cell.2008.05.034

[jcb25418-bib-0030] Palfy M , Remenyi A , Korcsmaros T . 2012 Endosomal crosstalk: Meeting points for signaling pathways. Trends Cell Biol 22:447–456. 22796207 10.1016/j.tcb.2012.06.004 PMC3430897

[jcb25418-bib-0031] Pawson T . 2004 Specificity in signal transduction: From phosphotyrosine‐SH2 domain interactions to complex cellular systems. Cell 116:191–203. 14744431 10.1016/s0092-8674(03)01077-8

[jcb25418-bib-0032] Platta HW , Stenmark H . 2011 Endocytosis and signaling. Curr Opin Cell Biol 23:393–403. 21474295 10.1016/j.ceb.2011.03.008

[jcb25418-bib-0033] Razidlo GL , Wang Y , Chen J , Krueger EW , Billadeau DD , McNiven MA . 2013 Dynamin 2 potentiates invasive migration of pancreatic tumor cells through stabilization of the Rac1 GEF Vav1. Dev Cell 24:573–585. 23537630 10.1016/j.devcel.2013.02.010 PMC3905678

[jcb25418-bib-0034] Rebsamen M , Pochini L , Stasyk T , de Araujo ME , Galluccio M , Kandasamy RK , Snijder B , Fauster A , Rudashevskaya EL , Bruckner M , Scorzoni S , Filipek PA , Huber KV , Bigenzahn JW , Heinz LX , Kraft C , Bennett KL , Indiveri C , Huber LA , Superti‐Furga G . 2015 SLC38A9 is a component of the lysosomal amino acid sensing machinery that controls mTO RC1. Nature 519:477–481. 25561175 10.1038/nature14107 PMC4376665

[jcb25418-bib-0035] Roepstorff K , Grandal MV , Henriksen L , Knudsen SL , Lerdrup M , Grovdal L , Willumsen BM , van Deurs B . 2009 Differential effects of EGFR ligands on endocytic sorting of the receptor. Traffic 10:1115–1127. 19531065 10.1111/j.1600-0854.2009.00943.x PMC2723868

[jcb25418-bib-0036] Rohatgi RA , Janusis J , Leonard D , Bellve KD , Fogarty KE , Baehrecke EH , Corvera S , Shaw LM . 2015 Beclin 1 regulates growth factor receptor signaling in breast cancer. Oncogene 34:5352–5362. 25639875 10.1038/onc.2014.454 PMC4522409

[jcb25418-bib-0037] Sadowski L , Pilecka I , Miaczynska M . 2009 Signaling from endosomes: Location makes a difference. Exp Cell Res 315:1601–1609. 18930045 10.1016/j.yexcr.2008.09.021

[jcb25418-bib-0038] Sancak Y , Bar‐Peled L , Zoncu R , Markhard AL , Nada S , Sabatini DM . 2010 Ragulator‐Rag complex targets mTORC1 to the lysosomal surface and is necessary for its activation by amino acids. Cell 141:290–303. 20381137 10.1016/j.cell.2010.02.024 PMC3024592

[jcb25418-bib-0039] Scheffler JM , Sparber F , Tripp CH , Herrmann C , Humenberger A , Blitz J , Romani N , Stoitzner P , Huber LA . 2014 LAMTOR2 regulates dendritic cell homeostasis through FLT3‐dependent mTOR signalling. Nat Commun 5:5138. 25336251 10.1038/ncomms6138 PMC4220488

[jcb25418-bib-0040] Schiefermeier N , Scheffler JM , de Araujo ME , Stasyk T , Yordanov T , Ebner HL , Offterdinger M , Munck S , Hess MW , Wickström SA , Lange A , Wunderlich W , Fässler R , Teis D , Huber LA . 2014 The late endosomal p14‐MP1 (LAMTOR2/3) complex regulates focal adhesion dynamics during cell migration. J Cell Biol 205:525–540. 24841562 10.1083/jcb.201310043 PMC4033770

[jcb25418-bib-0041] Schiefermeier N , Teis D , Huber LA . 2011 Endosomal signaling and cell migration. Curr Opin Cell Biol 23:615–620. 21546233 10.1016/j.ceb.2011.04.001 PMC3188704

[jcb25418-bib-0042] Sigismund S , Argenzio E , Tosoni D , Cavallaro E , Polo S , Di Fiore PP . 2008 Clathrin‐mediated internalization is essential for sustained EGFR signaling but dispensable for degradation. Dev Cell 15:209–219. 18694561 10.1016/j.devcel.2008.06.012

[jcb25418-bib-0043] Sorkin A , von Zastrow M . 2009 Endocytosis and signalling: Intertwining molecular networks. Nat Rev Mol Cell Biol 10:609–622. 19696798 10.1038/nrm2748 PMC2895425

[jcb25418-bib-0044] Stasyk T , Schiefermeier N , Skvortsov S , Zwierzina H , Peranen J , Bonn GK , Huber LA . 2007 Identification of endosomal epidermal growth factor receptor signaling targets by functional organelle proteomics. Mol Cell Proteomics 6:908–922. 17293594 10.1074/mcp.M600463-MCP200

[jcb25418-bib-0045] Taub N , Teis D , Ebner HL , Hess MW , Huber LA . 2007 Late endosomal traffic of the epidermal growth factor receptor ensures spatial and temporal fidelity of mitogen‐activated protein kinase signaling. Mol Biol Cell 18:4698–4710. 17881733 10.1091/mbc.E07-02-0098 PMC2096590

[jcb25418-bib-0046] Teis D , Taub N , Kurzbauer R , Hilber D , de Araujo ME , Erlacher M , Offterdinger M , Villunger A , Geley S , Bohn G , Klein C , Hess MW , Huber LA . 2006 P14‐MP1‐MEK1 signaling regulates endosomal traffic and cellular proliferation during tissue homeostasis. J Cell Biol 175:861–868. 17178906 10.1083/jcb.200607025 PMC2064696

[jcb25418-bib-0047] Teis D , Wunderlich W , Huber LA . 2002 Localization of the MP1‐MAPK scaffold complex to endosomes is mediated by p14 and required for signal transduction. Dev Cell 3:803–814. 12479806 10.1016/s1534-5807(02)00364-7

[jcb25418-bib-0048] Tsvetanova NG , Irannejad R , von Zastrow M . 2015 G protein‐coupled receptor (GPCR) signaling via heterotrimeric G proteins from endosomes. J Biol Chem 290:6689–6696. 25605726 10.1074/jbc.R114.617951 PMC4358092

[jcb25418-bib-0049] Verma N , Keinan O , Selitrennik M , Karn T , Filipits M , Lev S . 2015 PYK2 sustains endosomal‐derived receptor signalling and enhances epithelial‐to‐mesenchymal transition. Nat Commun 6:6064. 25648557 10.1038/ncomms7064

[jcb25418-bib-0050] Vilardaga JP , Jean‐Alphonse FG , Gardella TJ . 2014 Endosomal generation of cAMP in GPCR signaling. Nat Chem Biol 10:700–706. 25271346 10.1038/nchembio.1611 PMC4417940

[jcb25418-bib-0051] Vogel GF , Ebner HL , de Araujo ME , Schmiedinger T , Eiter O , Pircher H , Gutleben K , Witting B , Teis D , Huber LA , Hess MW . 2015 Ultrastructural morphometry points to a new role for LAMTOR2 in regulating the endo/lysosomal system. Traffic 16:617–634. 25677580 10.1111/tra.12271

[jcb25418-bib-0052] von Kleist L , Haucke V . 2012 At the crossroads of chemistry and cell biology: Inhibiting membrane traffic by small molecules. Traffic 13:495–504. 21951680 10.1111/j.1600-0854.2011.01292.x

[jcb25418-bib-0053] Wang S , Tsun ZY , Wolfson RL , Shen K , Wyant GA , Plovanich ME , Yuan ED , Jones TD , Chantranupong L , Comb W , Wang T , Bar‐Peled L , Zoncu R , Straub C , Kim C , Park J , Sabatini BL , Sabatini DM . 2015 Metabolism. Lysosomal amino acid transporter SLC38A9 signals arginine sufficiency to mTO RC1. Science 347:188–194. 25567906 10.1126/science.1257132 PMC4295826

[jcb25418-bib-0054] Waterman H , Sabanai I , Geiger B , Yarden Y . 1998 Alternative intracellular routing of ErbB receptors may determine signaling potency. J Biol Chem 273:13819–13827. 9593726 10.1074/jbc.273.22.13819

[jcb25418-bib-0055] Wolfson RL , Chantranupong L , Saxton RA , Shen K , Scaria SM , Cantor JR , Sabatini DM . 2015 Sestrin2 is a leucine sensor for the mTORC1 pathway. Science. 10.1126/science.aab2674 PMC4698017 26449471

[jcb25418-bib-0056] Worthylake R , Opresko LK , Wiley HS . 1999 ErbB‐2 amplification inhibits down‐regulation and induces constitutive activation of both ErbB‐2 and epidermal growth factor receptors. J Biol Chem 274:8865–8874. 10085130 10.1074/jbc.274.13.8865

[jcb25418-bib-0057] Zoncu R , Efeyan A , Sabatini DM. 2011 mTOR: From growth signal integration to cancer, diabetes and ageing. Nat Rev Mol Cell Biol 12:21–35. 21157483 10.1038/nrm3025 PMC3390257

